# Body Composition in Swiss Elite Wheelchair Athletes

**DOI:** 10.3389/fnut.2020.00001

**Published:** 2020-01-22

**Authors:** Joelle Leonie Flueck

**Affiliations:** Swiss Paraplegic Centre, Institute of Sports Medicine, Nottwil, Switzerland

**Keywords:** paralympic, anthropometry, spinal cord injury, dual X-ray absorptiometry, sport

## Abstract

The aim of this study was to retrospectively interpret body composition in various wheelchair athletes. In total, 69 athletes (mean ± standard deviation; age 33 ± 11 years; body mass 65.1 ± 14.8 kg; height 169.9 ± 14.9 cm and time since injury 19 ± 11 years) from different national teams in wheelchair sports underwent a dual X-ray absorptiometry (DXA) measurement during the yearly medical check-up. The data showed a significant difference between total fat and total fat-free mass between male (fat mass: 15.1 ± 7.6 kg; fat-free mass: 51.8 ± 9.3 kg) and female (fat mass: 19.4 ± 7.8 kg; fat-free mass: 36.8 ± 7.6 kg) athletes (*p* = 0.032, *p* < 0.001). In contrast, no significant difference (*p* = 0.16, *p* = 0.07) in fat and fat-free mass between paraplegic, tetraplegic and non-SCI athletes was found. Comparing different sports, the lowest fat mass was found in paracycling athletes whereas curling game players showed the highest total fat mass. Basketball game players showed the highest fat-free mass (fat-free mass: 54.8 ± 10.1 kg). In tetraplegic athletes, difference in fat-free mass between left and right arms correlated with the upper extremity motor score. For the interpretation of the data it seems to be crucial, that many different parameters (i.e., gender, motor level of the injury) are taken into consideration in wheelchair athletes.

## Introduction

It is already very well-known, that a chronic lesion of the spinal cord might lead to a significant loss of muscle mass in the paralyzed limbs and an increase in fat tissue due to immobility (1, 2). Thus, this generates a difference in body composition compared to healthy able-bodied individuals (3–5). On the other hand, it seems obvious, that exercise is effective in reducing total body fat content not only in able-bodied but also in individuals with a spinal cord injury (6, 7). It has been demonstrated, that periodization of body composition during a career is needed to optimize training adaptations and to guarantee the health of the athlete (8). Therefore, it seems worthwhile to track body composition in elite wheelchair athletes in order to optimize their energy needs regarding to their training schedule as well as to optimize performance in weight-dependent sports disciplines.

For measuring or estimating body composition, several different methods are already well-studied in able-bodied individuals (9–12). But, several of those methods show some limitations for detecting body composition in individuals with a spinal cord injury. One preferred method in able-bodied athletes is the dual X-ray absorptiometry (DXA) which distinguishes between fat, lean tissue and bone mineral content and calculates those values for different body compartments (9, 12–14). Goosey-Tolfrey et al. (15) compared several different methods to measure body composition in comparison to the DXA measurement in wheelchair games players. Their conclusion was, that air displacement plethysmography as well as existing skinfold equations and bio-impedance analysis should be used with caution in wheelchair athletes especially in athletes with substantial body asymmetry. Keil et al. (16) assessed test-retest reliability of a DXA measurement in elite wheelchair athletes. In this study, athletes were measured twice with a short break for repositioning in between the measurements. The least significant detectable change in wheelchair athletes was found to be 1.0 kg of fat mass, 1.1 kg of lean body mass and 0.12 kg of bone mineral content. The coefficient of variation (CV) ranged between 0.1 and 3.7% for the measurement of the compartments except for the fat mass content in the arms (CV = 7.8%). Thus, the authors concluded that this method might be valid and reliable to detect changes in body composition in wheelchair athletes. Another study showed that the precision of a DXA measurement in individuals with a chronic spinal cord injury is very similar to what was found in non-disabled individuals (17). To summarize, it is highly possible, that the DXA measurement is, for now, the appropriate method to use when working with individuals or athletes with a spinal cord injury.

Several different studies investigated body composition in wheelchair athletes using the DXA method (6, 15, 16, 18–20). Most of these studies focused on one sports discipline, a gender or one lesion level (e.g., tetraplegia or paraplegia). To our knowledge, no other study investigated body composition between wheelchair athletes from different sports. Therefore, the aim of this study was to illustrate DXA measurements of wheelchair athletes from different sports, impairment types and gender and to reflect what aspects might be of interest by interpreting those data.

## Materials and Methods

### Participants

In total, 69 wheelchair athletes (49 men and 20 women) participated in the study (Table 1). They were all member of a national team in a wheelchair sport, such as curling, paracycling (only handcycling athletes), wheelchair rugby, wheelchair basketball, wheelchair racing, table tennis, tennis, badminton, ski alpine, archery, and shooting. The study was approved by the local ethical committee [No. 2018-01738, Ethikkommission Nordwest-und Zentralschweiz (EKNZ), Basel, Switzerland].

**Table 1:** Characteristics of the athletes.

**Group**	***N***	**Age** **(years)**	**Body mass** **(kg)**	**Height** **(cm)**	**Time since injury (years)**
Paracycling	11	34 ± 11	60.5 ± 10.8	172.3 ± 10.1	19.3 ± 11.8
Rugby	14	31 ± 6	71.7 ± 18.5	178.1 ± 11.0	14.2 ± 8.4
Basketball	6	33 ± 10	70.2 ± 10.8	172.5 ± 12.0	25.8 ± 0.4
Athletics	13	26 ± 8	53.2 ± 12.0	161.8 ± 15.8	21.2 ± 10.8
Curling	6	51 ± 2	74.9 ± 8.9	172.7 ± 3.6	23.1 ± 10.2
Court sports	8	35 ± 15	64.0 ± 13.6	166.6 ± 11.1	19.8 ± 15.8
Others	10	32 ± 13	67.9 ± 12.5	164.7 ± 14.9	13.0 ± 5.3
Men	49	34 ± 11	68.9 ± 13.7	175.3 ± 9.7	19.0 ± 11.4
Women	20	32 ± 12	56.4 ± 14.1	156.7 ± 17.4	19.1 ± 7.3
Paraplegia	36	35 ± 13	62.9 ± 13.0	168.4 ± 12.6	21.2 ± 10.9
Tetraplegia	19	34 ± 8	71.5 ± 16.9	178.1 ± 9.1	15.5 ± 9.4
Non-SCI	14	30 ± 12	62.2 ± 14.7	162.4 ± 21.2	15.7 ± 0.9
Total	69	33 ± 11	65.1 ± 14.8	169.9 ± 14.9	19.0 ± 10.5

*SCI, spinal cord injury; court sports, table tennis, tennis, badminton; others, ski alpine, shooting and archery; N, number of participants; Non-SCI, amputees and cerebral palsy (wheelchair-dependent)*.

### Study Design

Data was retrieved retrospectively from the clinical information system. At the yearly medical check-up, a full-body DXA scan was performed. The medical check-up was performed 2–4 weeks before the first competition during the pre-season of each sports discipline. Body mass was measured in sitting position using a wheelchair scale (Busch BIT 650, Paul Busch Waagen Fabrik GmbH & Co., Hagen, Germany) wearing minimal clothing. The measurement took place before transferring onto the DXA device. Height was quantified in supine position using a measuring tape. Medical history data, such as the lesion level, the completeness as well as the duration of the lesion were recorded during the medical check-up. From the medical history, data of the upper extremity motor score (UEMS) and the motor level of the injury (MLI) were retrieved.

### Body Composition

Body composition was determined using a DXA device (Hologic, RRID:SCR_015529) with analysis performed using the Apex software (Hologic, RRID:SCR_015529). The scanner was tested for consistent calibration daily with phantoms used as per manufacturer guidelines each day for quality control purposes. All the scans were undertaken using the array mode. Scanning and analysis was performed by two trained radiographer who performed DXA measurements on a daily basis. A trained and experienced radiographer has positioned all participants as best as possible to obtain a valid measurement. In the case they had spasms during the scan they were repositioned and the scan was repeated. Athletes didn't performed any strenuous exercise on the day before the measurement. They were advised to have breakfast or lunch 2 h before the measurement and drink a glass of water with this meal as it was not possible to test them early in the morning in a fasted state. At the medical check-up, the last meal was self-recorded by the athlete. As they had to undergo a performance test afterwards, the athletes ate a carbohydrate-based, easy digestible meal 2 h before the DXA scan. The participants wore minimal clothing and the bladder was voided.

### Statistics

Statistical analysis was performed using the software IBM SPSS Statistics Version 23.0 (SPSS, RRID:SCR_002865). Statistical significance was set at the α-level of 0.05. Distribution of the data was tested by using the Kolmogorov-Smirnov, the Shapiro-Wilk test and the Q-Q plot. The mean ± standard deviation (SD) was used to describe the data.

The results indicated, that data was normally distributed for the whole group, as well as for gender (e.g., male vs. female) and injury group (e.g., tetraplegia, paraplegia, non-SCI). Therefore, the parametric *t*-test was used to detect significant differences between male and female participants as well as between participants with a tetraplegia and paraplegia. The Cohen's *d* as well as the effect size *r* were calculated.

Data in the different wheelchair sports subgroups were not normally distributed. Therefore, differences between the sports groups (i.e., paracycling, athletics, curling) were detected using non-parametric tests, such as the Kruskal-Wallis test and the Mann-Whitney-U test in case of significant findings between groups.

## Results

The 69 wheelchair athletes showed a total body fat content of 25.2 ± 9.5% and a fat content of 20.8 ± 10.7% in the arms, 22.5 ± 9.5% in the trunk and 35.1 ± 15.1% in the legs. The athletes showed a total fat mass of 16.3 ± 7.8 kg and a fat-free mass of 47.5 ± 11.2 kg. Fat-free and fat mass of different compartments (e.g., leg, arm, and trunk) for the different sports are displayed in Table 2. Significant findings were shown in the fat-free mass of the legs (right: *p* = 0.010, left: *p* = 0.001). Wheelchair rugby and basketball game players showed significantly higher fat-free mass in their legs compared to the other sports whereas curling game players showed significantly higher fat mass in the trunk compared to the other sports (*p* = 0.043). Comparing total fat-free (*p* = 0.08) and fat mass (*p* = 0.06) of the different sports, no significant differences were found (Table 3).

**Table 2:** Fat-free mass and fat mass in various different sports for three compartments.

**Group**	***N***	**Fat-free mass (kg)**			**Fat mass (kg)**		
		**Arms**	**Legs**	**Trunk**	**Arms**	**Legs**	**Trunk**
Paracycling	11	7.8 ± 2.0	10.1 ± 4.2	22.6 ± 3.4	1.4 ± 0.8	5.0 ± 2.8	5.2 ± 3.4
Rugby	14	7.0 ± 1.6	14.1 ± 4.1^*^	25.7 ± 5.5	1.9 ± 1.2	5.8 ± 2.9	8.0 ± 5.2
Basketball	6	9.0 ± 1.0	13.1 ± 5.5^*^	27.6 ± 4.4	1.7 ± 0.9	4.6 ± 1.9	7.4 ± 4.7
Athletics	13	6.6 ± 2.4	6.9 ± 3.8	20.5 ± 6.2	1.8 ± 0.8	5.4 ± 2.4	5.8 ± 2.4
Curling	6	7.6 ± 1.5	10.4 ± 2.8	26.0 ± 3.3	2.9 ± 0.9	8.7 ± 2.4	12.7 ± 3.8^*^
Court sports	8	6.3 ± 1.4	10.4 ± 3.9	22.4 ± 5.0	2.5 ± 0.9	7.1 ± 3.8	7.8 ± 4.8
Others	10	7.2 ± 2.0	10.5 ± 5.3	24.7 ± 5.3	2.2 ± 1.0	6.5 ± 2.8	7.8 ± 3.5
Total	69	7.2 ± 1.9	10.7 ± 4.8	23.9 ± 5.3	2.0 ± 1.1	6.0 ± 2.9	7.5 ± 4.4

Court sports, table tennis, tennis, badminton; others, ski alpine, shooting and archery; N, number of participants;

* *Significantly different to other sports (p < 0.05)*.

**Table 3:** Fat-free and fat mass in different sports.

**Group**	***N***	**Fat-free mass (kg)**	**Fat mass (kg)**
Paracycling	11	46.1 ± 8.3	12.5 ± 6.5
Rugby	14	52.5 ± 11.5	16.7 ± 9.1
Basketball	6	54.8 ± 10.1	14.2 ± 7.8
Athletics	13	39.7 ± 11.8	14.4 ± 5.0
Curling	6	49.9 ± 7.0	25.3 ± 4.9
Court sports	8	44.5 ± 9.9	18.3 ± 9.9
Others	10	48.0 ± 11.4	17.1 ± 6.8

*Court sports, table tennis, tennis, badminton; others, ski alpine, shooting and archery; N, number of participants*.

**Figure 1:**
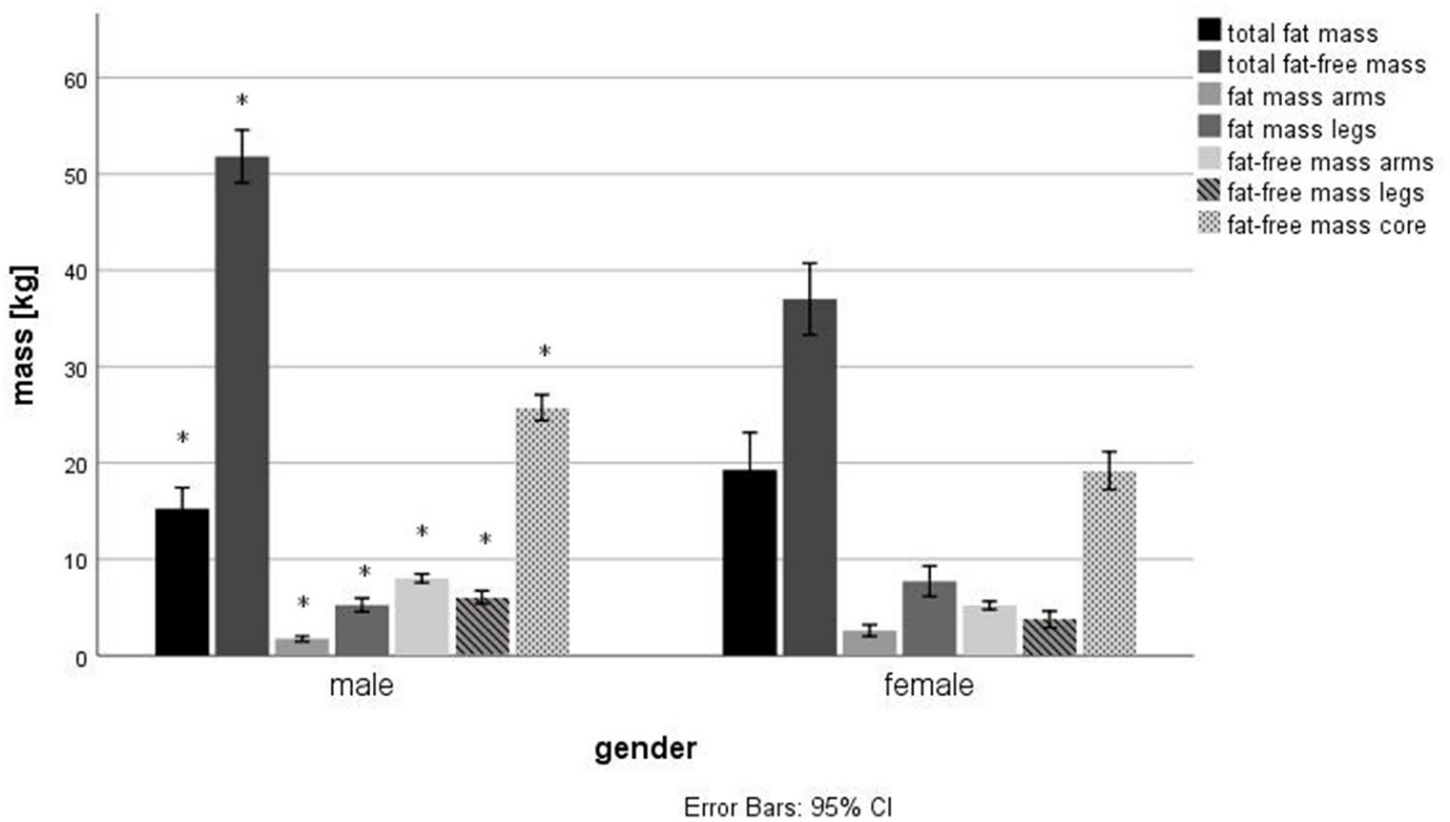
Differences in body composition in male and female wheelchair athletes. *Significant difference (*p* < 0.05) between male and female.

**Figure 2:**
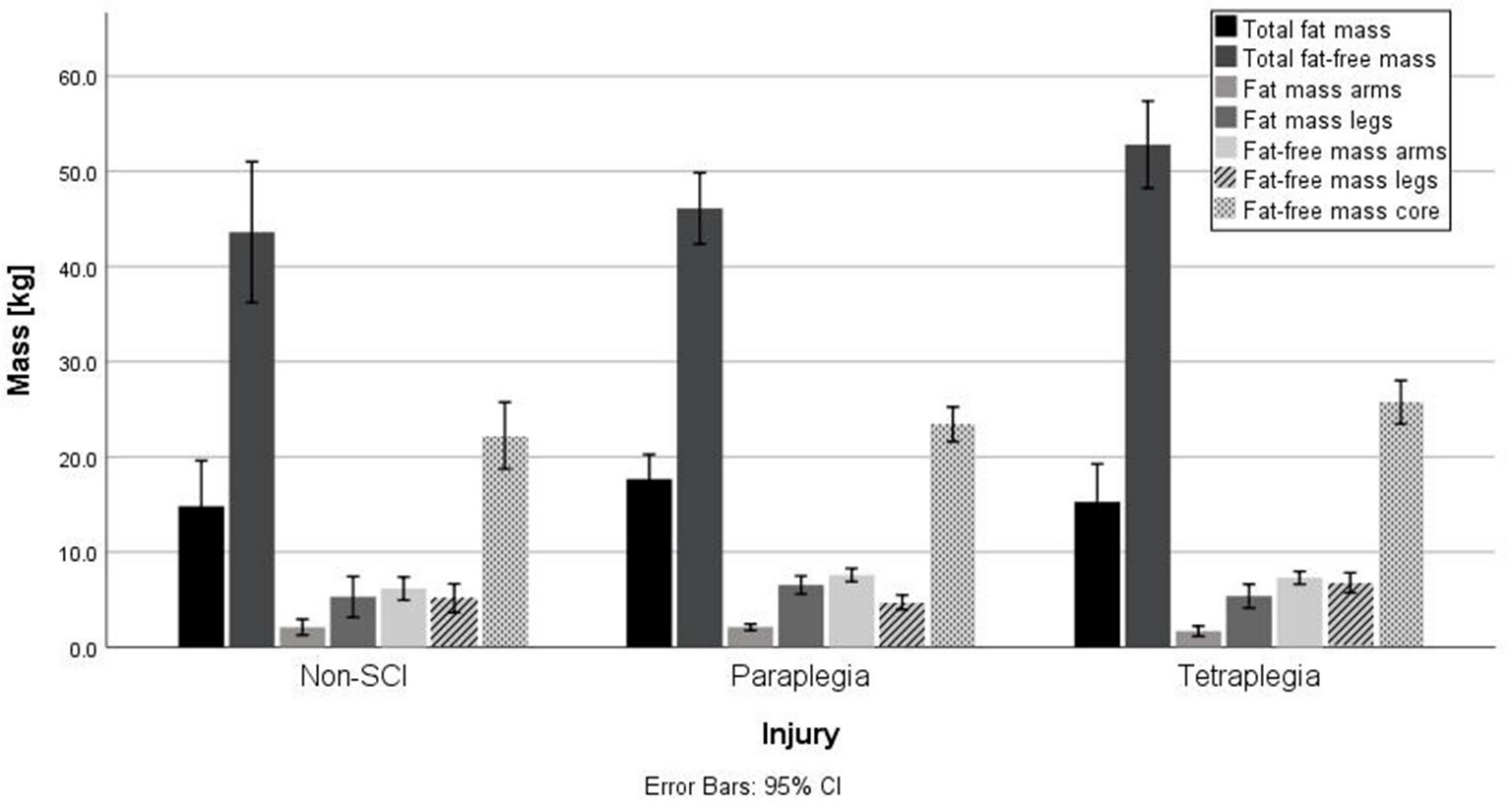
Differences in body composition in paraplegic, tetraplegic and non-SCI athletes. No significant differences were found.

**Figure 3:**
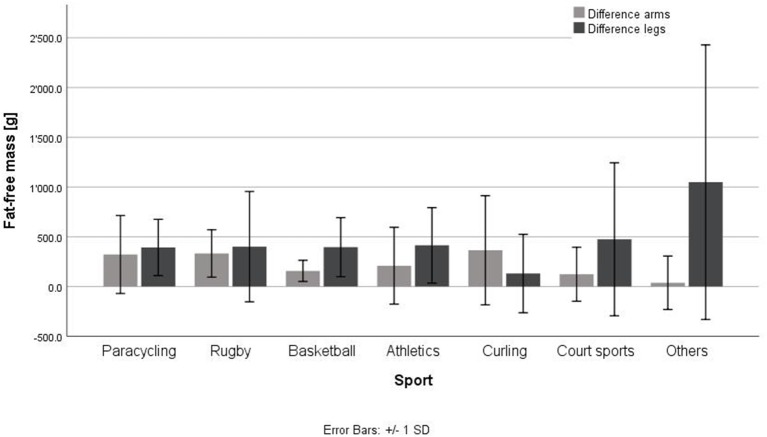
Differences in fat-free mass between left and right arm and leg in different wheelchair sports. Court sports, tennis, badminton, table tennis; others, ski alpine, shooting, archery, no significant differences were found.

Figures 1, 2 show the fat and fat-free mass in male and females as well as in tetraplegic, paraplegic and non-SCI athletes. Total fat and fat-free mass were significantly different between male and female athletes (*p* < 0.001, FM: *d* = 0.56, *r* = 0.27; FFM: *d* = 1.77, *r* = 0.66). Significant differences were detected in all parameters (*p* < 0.05) except for difference in fat-free mass in the arms (*p* = 0.40), difference in fat-free mass in the legs (*p* = 0.31) and in the fat mass of the trunk (*p* = 0.42). Females (Total: 33.6 ± 7.0%, arms: 31.6 ± 9.7%, trunk: 28.2 ± 8.4%, legs: 50.1 ± 10.1%) showed a significantly higher fat percentage in all compartments (*p* < 0.05, total: *d* = 1.55, *r* = 0.61; arms: *d* = 1.76, *r* = 0.66; trunk: *d* = 0.93, *r* = 0.42; legs: *d* = 1.90, *r* = 0.69) compared to males (Total: 21.8 ± 8.2%, arms: 16.4 ± 7.5%, trunk: 20.1 ± 9.0%, legs: 28.8 ± 12.3%). Total fat and fat-free mass were not significantly (*p* = 0.16, *p* = 0.07) different between tetraplegic, paraplegic, and non-SCI athletes. Paraplegic athletes showed a significantly lower fat-free mass (*p* < 0.001, *d* = 0.69, *r* = 0.32) and a significantly higher fat percentage (*p* < 0.001, *d* = 1.36, *r* = 0.56) in the legs compared to tetraplegic athletes. Differences between left and right arm (*p* = 0.60) as well as between left and right leg (*p* = 0.89) of fat-free mass of the different sports are shown in Figure 3. The difference between arms was 230 ± 333 and 473 ± 687 g between legs over all participants. Table 4 shows the differences between left and right side of the upper extremity in completely and incompletely lesioned tetraplegic athletes. In most cases, the arm with the higher fat-free mass showed also a higher UEMS. Paraplegic athletes showed a total difference between the arms of 195 ± 297 g whereas non-SCI athletes showed a difference of 210 ± 413 g. The difference in fat-free mass between the right and the left arm was significantly higher in tetraplegic athletes compared to paraplegic athletes (*p* = 0.013, *d* = 0.37, *r* = 0.18). A comparison between the body mass on the scale (65.1 ± 1.5 kg) and the body mass determined through the DXA scan (63.8 ± 1.5 kg) revealed a significant difference (*p* = 0.001).

**Table 4:** Difference between left and right side of upper extremity in tetraplegic athletes in comparison to motor level of lesion.

**Participant**	**Lesion level**	**MLI**		**Sport**	**Fat-free mass (kg)**		**Difference (g)**	**UEMS**		**Dominant side**	**Congruence**
**Incomplete lesion**		**Right**	**Left**		**Right arm**	**Left arm**	**Right vs. Left**	**Right**	**Left**		
1	C2	C5	C3	Athletics	3.06	2.43	636.3	20	14	Right	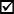
2	C4	C6	C6	Rugby	2.63	2.75	112.0	11	11	No	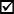
3	C5	C5	C7	Paracycling	3.65	4.10	450.3	14	20	Left	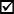
4	C5	C6	C6	Rugby	2.61	2.47	140.1	9	16	Left	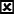
5	C5	n.a.	n.a.	Rugby	4.82	4.28	540.2	n.a.	n.a.	n.a.	n.a.
6	C5	C7	C7	Rugby	4.21	3.83	372.5	23	20	Right	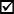
7	C5	C7	C6	Athletics	3.39	2.79	598.0	19	13	Right	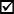
8	C5	C6	C5	Athletics	5.35	5.37	17.6	20	19	No	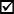
9	C6	C7	C8	Rugby	2.86	3.01	141.1	18	23	Left	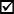
10	C6	C8	C7	Rugby	4.81	4.18	627.7	24	23	Right	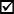
11	C6	C8	C7	Rugby	3.33	3.02	307.8	19	15	Right	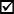
12	C6	n.a.	n.a.	Rugby	3.73	3.47	256.2	n.a.	n.a.	n.a.	n.a.
13	C6	n.a.	n.a.	Rugby	4.70	3.85	843.9	n.a.	n.a.	n.a.	n.a.
14	C6	n.a.	n.a.	Rugby	4.38	4.15	238.1	n.a.	n.a.	n.a.	n.a.
15	C7	C7	C7	Athletics	2.88	3.70	824.2	19	19	No	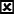
16	C8	C8	C8	Rugby	3.75	3.70	53.2	22	19	Right	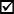
**Mean ± SD**					**3.76 ± 0.87**	**3.57 ± 0.79**	**305.4 ± 361.6**	**18.1 ± 4.6**	**17.7 ± 3.8**		
**Complete lesion**											
17	C6	C6	C6	Paracycling	3.87	3.84	28.5	11	14	Left	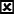
18	C6	T1	C8	Paracycling	3.39	2.97	415.4	24	19	Right	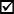
19	C6	C6	C6	Rugby	3.83	3.18	643.9	10	10	n.a.	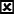
20	C7	C7	C5	Rugby	3.84	3.58	262.7	24	16	Right	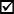
**Mean ± SD**					**3.39 ± 0.39**	**3.73 ± 0.23**	**337.6 ± 258.9**	**17.2 ± 6.7**	**14.8 ± 3.8**		

*No statistical significance occurred, UEMS, upper extremity motor score; MLI, motor level of injury; n.a., not applicable; no, no dominant side; congruence, UEMS and fat-free mass showed the same dominant side. Bold values indicates mean ± SD of all participants above*.

## Discussion

This data analysis aimed to illustrate the body composition in various disciplines of wheelchair sports. Female athletes showed a significantly lower fat-free mass compared to male athletes, whereas fat mass was significantly higher. Interestingly, tetraplegic athletes showed often a dominant side dependent on their injury that was expressed by a higher fat-free mass in the arms in this specific side (Table 4).

### Body Composition in Able-Bodied and Disabled Athletes

Endurance sports athletes (e.g., paracycling, wheelchair racing) showed the lowest body mass from all sports. Total fat mass was the lowest in paracycling athletes although not significantly lower compared to other sports. Comparing the 12.5 ± 6.5 kg fat mass of paracycling athletes to the 25.3 ± 4.9 kg fat mass of curling players there seem to exist a sport specific influence on total fat mass (Table 3). Comparing fat-free mass in various sports disciplines, again no significant difference occurred (*p* = 0.08). This might have been influenced by the small sample size in each sports discipline. With a higher number of athletes per sport, possibly, significant differences would have occurred. In this case, a clinically relevant higher fat-free mass was described in basketball players. Endurance sports athletes showed a lower fat-free mass compared to other sports which is of course dependent from the lower total body mass. In general, fat percentage was not significantly different between the various sports disciplines. It seemed, that athletes with a higher fat percentage in the legs showed a lower fat-free mass in the legs. Leg mass contributes to a big part to total body mass and therefore, body composition from the legs could influence the total body fat percentage in those athletes. Therefore, it seems worthwhile to analyze fat mass and fat-free mass especially in the different compartments (Table 2). Spasticity might also have an influence on fat and fat-free mass in the legs by maintaining more muscle mass due to spasticity. Thus, the sports specific position in the wheelchair as well as the occurrence of spasticity might be of interest when assessing body composition in wheelchair athletes in the future.

Another finding concerned the contralateral differences in fat-free mass between left and right body side (Figure 3, Table 4). No significant differences between the various sports occurred in fat-free mass between left and right arms as well as left and right legs. Comparing the lesion level, athletes with a tetraplegia showed the highest difference in fat-free mass in the arms. In Table 4 it was shown that those differences could be explained by either the MLI as well as by the UEMS. Those data revealed a higher fat-free mass on the side with the higher UEMS. In personal discussions with the athletes, they reported very often, that one side had specifically less motor function and therefore, they use more the other side for specific tasks. In a group of 16 subjects with a cervical lesion level, it was shown, that most subjects reported a stronger side and differences in maximal voluntary contraction between contralateral sides in upper extremities (21). Furthermore, a laterality toward one body side was seen in individuals with tetraplegia during activity (22). Thus, it seems important to have a look for UEMS and MIL in the medical history of athletes with a tetraplegia before interpretation of the results.

Comparing fat-free mass in the arms, basketball players showed the highest followed by paracycling athletes, even though, there was no significant difference between the different sports disciplines (Table 2). Comparing these results to the study of Keil et al. (16), both studies showed similar results for fat mass and fat-free mass in the arms, legs and trunk in basketball players. The same was true for Willems et al. (19) investigating body composition in 14 wheelchair games player. Additionally, they showed a significantly lower total lean tissue mass in non-walkers compared to walkers.

Looking at female athletes alone, these data showed similar findings in comparison to other female wheelchair athletes (18). Body composition from female wheelchair athletes were compared to a reference group of female non-wheelchair athletes. Sutton et al. (18) found also a significantly higher fat percentage in the legs of wheelchair athletes, although fat mass in the legs was similar in both groups. The results again show that total body fat percentage might be influenced by the fat mass from the legs using other methods, where separate analysis of different body compartments is not possible. These authors concluded, that a DXA measurement might be a good standard method in wheelchair athletes (18). Cavedon et al. (23) compared male and female wheelchair basketball players and found significant differences in body fat percentage when assessed with the skinfold method. Those results are in line with our results whereas female athletes showed a higher body fat percentage compared to male athletes. In comparison to Pelly et al. (20), it seems that their athletes showed a lower fat-free mass and a lower fat mass. It is worth mentioning that they have tested only six athletes which might be not representative for a comparison with 69 athletes in this study.

Santos et al. (24) published reference values for various different sports disciplines in able-bodied athletes. They derived data from almost 500 DXA measurements. Comparing those values for total fat percentage, able-bodied athletes showed a significantly lower total fat percentage compared to our wheelchair athletes in their respective sports discipline. Loss of muscle function in lower as well as in upper extremities (i.e., tetraplegia) and an increase in fat mass due to immobilization or a positive energy balance might explain those differences (25). Therefore, it seems worthwhile to consider segmental body composition (i.e., upper body) through a DXA measurement when trying to compare body composition of wheelchair athletes to able-bodied athletes for similar sports disciplines (i.e., sports with upper body exercise).

### Body Composition in Individuals With a Spinal Cord Injury

It seems interesting, that athletes with a tetraplegia in this study showed a significantly lower fat mass in the legs as well as a significantly higher fat-free mass in the legs compared to paraplegic athletes. This fact results in the end in a significantly higher fat percentage in para- compared to tetraplegic athletes. Similar findings were shown by Singh et al. (26) whereas patients with a para- and tetraplegia in the first year following the incident of the injury were compared. Additionally, they showed a higher fat-free mass in the arms of individuals with paraplegia compared to tetraplegia. This might result from the motor lesion level (i.e., MLI, UEMS) and the impairment of the upper extremities in patients with tetraplegia. In general, the wheelchair athletes showed a higher fat-free mass compared to the patients in the study of Singh et al. (26) which results from strength or sports specific training. Additionally, Singh et al. (26) suggested a decrease in bone mineral content and lean body mass in the first year following the incident of the injury which leads to an increase in adiposity. Another study comparing body composition of participants with a chronic para- and tetraplegia showed in total a lower body fat percentage (34%) in paraplegia compared to tetraplegia (38%) (27). Although, total fat percentage was not significantly different in this study between athletes with para- or tetraplegia, a tendency (*p* = 0.051) of a higher fat percentage in paraplegia was found. This might result from the significantly higher fat percentage found in the legs of athletes with paraplegia (Figure 2). However, it might also be a result of a difference in training status, energy balance as well as level of physical activity in general. Similar findings were shown by McDonald et al. (28), when comparing patients with tetra- and paraplegia with a traumatic spinal cord injury to controls without spinal cord injury. They found a significantly higher body fat percentage in paraplegia (31%) vs. control (26%) as well as in paraplegia (31%) vs. tetraplegia (23%). A lower total lean body mass in individuals with a spinal cord injury compared to able-bodied controls was found (3, 4, 29). Fat mass revealed a good correlation with the duration of the injury (30, 31). Furthermore, the total body fat percentage was compared to the Body-Mass-Index (BMI), whereas McDonald et al. (28) suggested that BMI underestimated obesity in this population. Jones et al. (25) suggested, that the DXA scan might be the most appropriate measurement for body composition in individuals with a spinal cord injury. They recommend, that DXA scans should be used more often in order to prevent patients from adiposity and to track changes in body composition over the years.

### Limitations

It is recommended to conduct a DXA scan in a fasted and standardized hydrated state (12). In this study, it was not possible to ensure a fasted state as the athletes were tested during the yearly medical check-up and those data were analyzed retrospectively. Nevertheless, it was recommended to ingest the last meal 2 h before the measurement and to stay well-hydrated (32). No standardization of positioning aids was possible due to variety of differences in the athletes, but the scans were all performed with the same device and the same software. Two trained technicians performed the DXA measurement but as both of them perform those analyses on a regular daily basis, no quality problems should have occurred. The data from this study showed also a significant difference in body mass measured on scale and the one measured during the DXA scan. Such an error must be taken into account when interpreting such data. Furthermore, the time point of the testing was not standardized as the medical check-up took place according to the athletes' availability, but most of the testing was conducted during pre-season of the specific sports discipline. Thus, seasonal variations in body composition might play a role when trying to define reference values in the future (33). Additionally, comparing different sports including different types of injury (i.e., lesion level, completeness, and time of injury) seems to be a factor with a huge influence on body composition. It seems important to gain as much information about the athletes' injury before the interpretation of any data. Moreover, it was reported that the body composition characteristics of different players in a team sport might differ according to playing position (34). Due to a low sample size, it was not possible to compare body composition according to playing position in wheelchair team sports. In addition, the small sample size in each different sports might be a limitation for a proper data analysis. Additionally, the scope of the training, as well as the energy balance were not assessed in this data analysis, even though they will have a huge impact on body composition. Taking into account all of those limitations, it is somewhat impossible to define reference values for body composition in different wheelchair sports. Furthermore, it seems important to collect further data of body composition in wheelchair sports with more data per sports discipline, more standardization in the methods as well as for the time point of the measurement as well as more information on trainings scope and nutrition.

### Conclusions

This data showed that some sports specific differences between the body composition of various wheelchair sports and impairment types might occur. A DXA measurement seems to be a useful tool as body compartments can be analyzed separately. For the interpretation of the data it seems to be crucial, that many different parameters are taken into consideration in wheelchair athletes. Factors, such as gender, age or training level might be important in a first step. But taking into account the lesion level, the sensory and motor completeness as well as the duration of the injury, the laterality of muscle function and the differences in sports activities might be important for interpretation and optimization of those measurements.

## Data Availability Statement

The datasets generated for this study are available on request to the corresponding author.

## Ethics Statement

The studies involving human participants were reviewed and approved by EKNZ, Ethikkommission Nordwest-und Zentralschweiz, Basel, Switzerland. The patients/participants provided their written informed consent to participate in this study.

## Author Contributions

JF designed the study, collected the data, and wrote the publication.

### Conflict of Interest

The author declares that the research was conducted in the absence of any commercial or financial relationships that could be construed as a potential conflict of interest.
